# Phase II trial of prophylactic hyperthermic intraperitoneal chemotherapy in patients with locally advanced gastric cancer after curative surgery

**DOI:** 10.1186/s12885-021-07925-2

**Published:** 2021-03-02

**Authors:** Biao Fan, Zhaode Bu, Ji Zhang, Xianglong Zong, Xin Ji, Tao Fu, Ziyu Jia, Yinan Zhang, Xiaojiang Wu

**Affiliations:** Key Laboratory of Carcinogenesis and Translational Research (Ministry of Education), Gastrointestinal Cancer center, Peking University Cancer Hospital and Institute, Fu-Cheng Road 52#, Hai-Dian District, Beijing, 100142 China

**Keywords:** Locally advanced gastric cancer, HIPEC, Cisplatin, Safety, Clinical efficacy

## Abstract

**Background:**

HIPEC is an emerging procedure to treat peritoneal metastasis of gastric cancer. Data about HIPEC in locally advanced gastric cancer is scarce. The purpose of this trial is to evaluate the safety and toxicity of prophylactic HIPEC with cisplatin for patients with locally advanced gastric cancer.

**Methods:**

From March 2015 to November 2016, a prospective, randomized phase II trial was conducted. After radical gastrectomy, patients in the experimental group underwent HIPEC with cisplatin followed by adjuvant chemotherapy with SOX regime. Patients in the other group were treated with SOX regime alone. Postoperative complications and patient survival were compared.

**Results:**

In total, 50 patients were eligible for analyses. No significant difference was found in the incidence of postoperative complications including anastomotic/intestinal leakage, liver dysfunction, bone marrow suppression, wound infection and ileus (*P* > 0.05). Mean duration of hospitalization after radical gastrectomy was 11.7 days. 12.2 days in experimental group and 10.8 days in control group respectively (*P* = 0.255). The percentage of patients with elevated tumor markers was 12.1% in experimental group, which was significantly lower than 41.2% in control group (*P* = 0.02). 3-year RFS of patients who treated with or without prophylactic HIPEC were 84.8 and 88.2% respectively (*P* = 0.986). In the multivariate analysis, pathological T stage was the only independent risk factor for the RFS of patients (*P* = 0.012, HR =15.071).

**Conclusion:**

Additional intraoperative HIPEC with cisplatin did not increase postoperative complications for locally advanced gastric cancer after curative surgery. Prophylactic HIPEC with cisplatin was safe and tolerable, while it did not reduce the risk of peritoneal recurrence in this trial, supporting further studies to validate the efficacy of it.

**Trial registration:**

Chinese Clinical Trial Registry, ChiCTR2000038331. Registered 18 September 2020 - Retrospectively registered, http://www.chictr.org.cn/showproj.aspx?proj=59692.

## Background

Gastric cancers is one of the most common cancer in the world, especially in Asian countries such as China, Japan and Korea [1, 2]. Peritoneal recurrence is a common pattern of gastric cancer recurrence, and it often indicates poor prognosis [3–5]. When compared with lymphatic and hematogenous metastasis, peritoneal metastasis is considered as a local disease that could be treated by HIPEC. HIPEC is an effective procedure for peritoneal metastasis of multiple cancers, including ovarian cancer and appendix mucinous adenocarcinoma, by which a high concentration of chemotherapeutic drug can directly act on peritoneal cancer cells and nodules [6–9]. There is also some clinical evidence supporting the efficacy of HIPEC for peritoneal metastasis of gastric cancer [10–13].

For the locally advanced gastric cancer, without non-curative factors, the most widely accepted therapies are neoadjuvant chemotherapy and radical gastrectomy plus D2 lymphadenectomy followed by adjuvant chemotherapy. Based on the seed and soil theory, peritoneal metastasis is defined as the implantation and progression of cancer cells seeded on peritoneum [14–16]. Studies have demonstrated that peritoneal micro-metastasis was detected in 5–20.5% of patients with locally advanced gastric cancer [17–19]. Serosal invasion in gastric cancer was positively correlated with positive peritoneal wash cytology, which was thought to be the early stage of macroscopic peritoneal metastasis [20, 21]. After radical surgery, 43–45.9% of gastric cancer patients will suffer from peritoneal recurrence [22, 23].

The use of HIPEC is considered as a potential prophylactic approach to avoid peritoneal recurrence of cancer. Nevertheless, data about prophylactic HIPEC in gastric cancer is scarce, and it is difficult to outline its role against peritoneal recurrence. In this study, we evaluated the post-operational complications and outcomes of gastric cancer patients who underwent radical gastrectomy followed by HIPEC with cisplatin, focusing our analysis on the safety and tolerability of the experimental treatment, which could influence patient survival.

## Methods

### Study design and patients

This was a prospective, randomized, single center study conducted from March 2015 to November 2016. Patients with locally advanced gastric cancer (cT3-4 N0-3 M0) treated with radical gastrectomy at the Gastrointestinal Cancer Center of Peking University Cancer Hospital & Institute were enrolled. The clinical stage of patients was based on radiology results. There was no distant metastasis such as liver metastasis, lung metastasis and peritoneal metastasis in all patients. Diagnostic laparoscopy revealed that they all had negative cytology. According to the preliminary data, the peritoneal recurrence rate is 35% within 1 year after radical surgery in locally advanced gastric cancer patients treated without HIPEC (the control group proportion: 0.35), while that is 10% in patients treated with HIPEC (the experimental group proportion: 0.10). In this trial, the allocation ratio of patients in the experimental group and the control group is 2:1. The experimental group proportion is assumed to be 0.32 under the null hypothesis and 0.10 under the alternative hypothesis. The test statistic used is the two-sided Z test (unpooled). Using PASS 11, the required sample size should be 116 patients (77 patients in the experimental group and 39 patients in the control group), with a two-sided alpha level of 5% and a power of 80%. However, the trial was prematurely terminated after 50 patients enrolled, owing to the end of cooperation with the company that provided the HIPEC system (BR-TRG-II). This study was approved by the Ethics committee of Peking University Cancer Hospital. Written informed consent was obtained from all patients. The study adhered to CONSORT guidelines and it was registered at the Chinese Clinical Trial Registry (ChiCTR2000038331).

### Randomization and procedure of HIPEC

After radical gastrectomy, patients were randomly divided into two groups (a two-to-one ratio), namely, the experimental group (included patients who were treated with HIPEC followed by adjuvant chemotherapy with SOX regime) and the control group (included patients who underwent adjuvant chemotherapy with SOX regime alone). Randomization was performed via a computer generated sequence. In the experimental group, HIPEC was performed under general anesthesia after radical gastrectomy. Two outlet pipes and one inlet pipe were installed. Cisplatin (50 mg/L) was diluted in heated 0.9% sodium chloride and then circulated for 30 min. Perfusion rate was 400–500 ml/min. The circulation temperature was 42.5 °C - 43.0 °C. HIPEC perfusion and circulation was performed with BR-TRG-II (Bright Medical Technology Co.,Ltd., Guangzhou, China). After the HIPEC was finished, at least 90% of perfusion fluid was removed.

### Procedure of adjuvant chemotherapy

Pathologically, all patients with locally advanced gastric cancer received postoperative adjuvant chemotherapy (a 3 week cycle SOX regime). S-1, 40-60 mg (40 mg when BSA < 1.25 m^2^, 60 mg when BSA > 1.5 m^2^), twice per day, Day 1–14; Oxaliplatin (130 mg/m^2^) was given intravenously at the first day of each cycle. Toxicity of the regime was graded based on the Common Terminology Criteria for Adverse Events version 4.0.

### Clinical parameters and postoperative follow-up

Clinico-pathological parameters including tumor size, tumor differentiation, TNM stage, hospitalization day after surgery, drainage tube removal time and postoperative complications such as anastomotic leakage were prospectively recorded and analyzed. The primary endpoint was 3-year RFS. The secondary endpoint was the incidence of postoperative complications (based on Clavien-Dindo classification).

### Statistical analysis

Incidence of postoperative complications and patient outcome were compared. All statistical analyses were performed by using SPSS Statistics software version 17.0. Clinico-pathological characteristics and incidence of postoperative complications in both groups were compared via a two-tailed independent-sample t-test or a nonparametric tests. The Kaplan–Meier analysis was performed to estimate survival rates. Multivariate analysis was used for survival analysis. A two-sided *P* < 0.05 was taken as significant.

## Results

### Baseline data

In total, 50 patients (median age, 61 years old; range, 46–86 years old) from March 2015 to November 2016 were enrolled. Median follow-up was 37 months. Of these patients, 33 patients were performed HIPEC with cisplatin, the others underwent adjuvant SOX regime alone. Patients in this study were well balanced and comparable regarding baseline clinic-pathological parameters (*P* > 0.05; Table 1).

**Table 1:** Clinico-pathological parameters of fifty locally advanced gastric cancer patients treated with/without prophylactic HIPEC

	Control group (*n* = 17)	Experimental group (*n* = 33)
Age	60 (50–86)	61 (46–78)
Sex, M/F	14/3	27/6
KPS, 90/100	9/8	18/15
BMI	21 (15–32)	23 (16–34.5)
Disease history, Yes/No	11/6	13/20
Location, U/M/L	5/3/9	14/4/15
Size, < 5 cm/≥5 cm	13/4	16/17
Gastrectomy, Sub. /Tot.	10/7	15/18
Histological diagnosis, Well/intermediately versus poorly/undifferentiated	10/7	11/12
Lauren classification, Intestinal/diffuse/mixed	6/6/4	13/6/12
Lymphovascular invasion, Yes/No	6/11	18/15
Neurovascular invasion, Yes/No	9/8	15/18
T stage, 1 ~ 3/4	12/5	22/11
N stage, 0/1 ~ 3	10/7	10/23
Ki67 IHC	75% (50–99%)	60% (10–90%)
MSS/MSI	10/2	26/2

*KPS* karnofsky performance score; *BMI* body mass index; *MSS/MSI* micro-satellite stable/instable

### Tumor markers changing tendency and adverse events

The percentage of patients with elevated tumor markers was 12.1% in experimental group, which was significantly lower than in control group (41.2%; *P* = 0.02). There was no statistically significant difference between the occurrence rates of postoperative complications (*P* > 0.05; Table 2). Anastomotic or intestinal leakage occurred in 5 patients, 1 in control group (5.9%) and 4 in experimental group (12.1%) (*P* = 0.49). Three grade II liver dysfunction developed in control group, while 8 in experimental group (*P* = 0.773). Four patients showed grade II bone marrow suppression, 2 in each group. Neither wound infection nor ileus occurred in either groups. Mean duration of hospitalization after radical gastrectomy was 11.7 days. 12.2 days in experimental group and 10.8 days in control group respectively (*P* = 0.255). Mean drainage tube removal time after surgery was 9.7 days. 10.2 days in experimental group and 8.6 days in control group respectively (*P* = 0.159).

**Table 2:** Postoperative complications in patients treated with/without prophylactic HIPEC

	Control group (*n* = 17)	Experimental group (*n* = 33)	*P* value
Alb (< 35 g/L/≥35 g/L)	15/2	25/8	0.069
Change of tumor markers, Decrease/stable/increase	1/9/7	6/23/4	0.020
Median hospitalization day after surgery	10 (7–22)	11 (8–24)	0.255
Median drainage tube removal time after surgery	8 (6–17)	9 (5–24)	0.159
Anastomotic leakage/Intestinal leakage, Yes/No	1/16	4/29	0.490
Wound infection/Poor wound healing, Yes/No	0/17	0/33	1.000
Ileus, Yes/No	0/17	0/33	1.000
Leukopenia, None/grade I/II	13/2/2	25/6/2	0.956
Neutropenia, None/grade I/II	13/2/2	28/3/2	0.453
Liver dysfunction, None/grade I/II	4/10/3	10/15/8	0.773

*Alb* Albumin

### Survival and recurrence

**Fig. 1:**
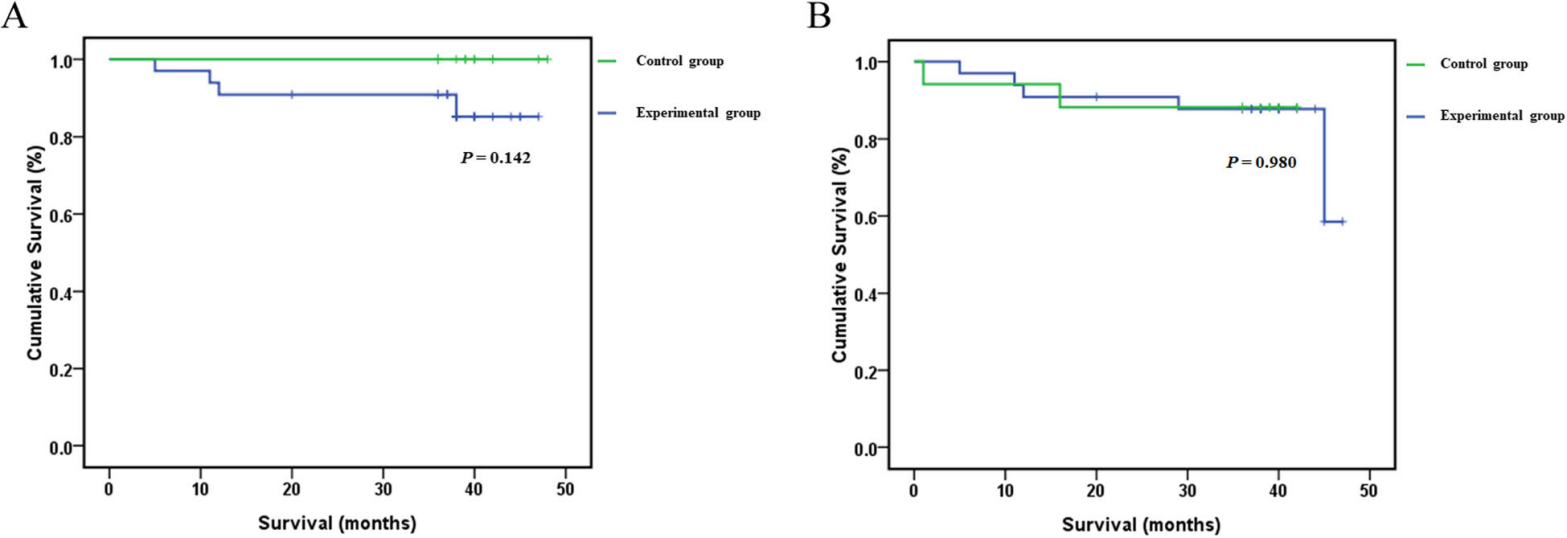
Survival of patients with locally advanced gastric cancer after radical gastrectomy. **a** 3-year overall survival; **b** 3-year relapse-free survival

**Fig. 2:**
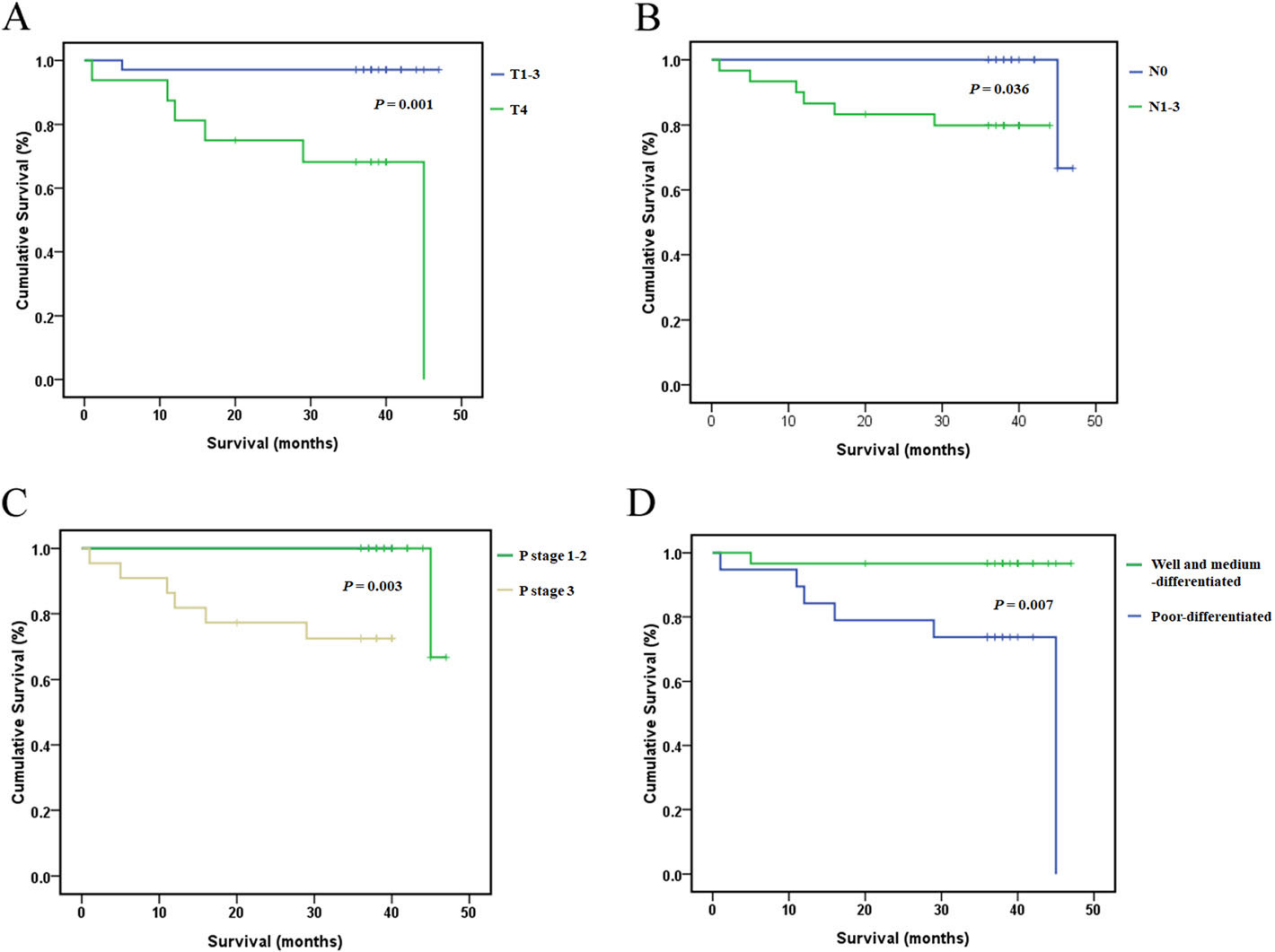
Impact of tumor stage and tumor differentiation on patient relapse-free survival. **a** T stage; **b** N stage; **c** pTNM stage; **d** Tumor differentiation

The follow-up time ranged from 36 to 48 months (median: 37 months). 3-year overall survival rate of the whole cohort was 92, 87.9% in experimental group and 100% in control group respectively (*P* = 0.142; Fig. 1a). The 3-year RFS rates of patients who underwent HIPEC or not were 84.8 and 88.2% respectively (*P* = 0.980; Fig. 1b). Prognostic factors were pathological T stage, N stage, pTNM stage and tumor differentiation by univariate analysis (*P* < 0.05; Fig. 2a-d). Multivariate analysis of RFS showed that only pathological T stage (RR 15.071, *P* = 0.012) was significantly associated with prognosis (Table 3).

**Table 3:** Multivariate predictors of survival

Variables	Risk ratio	95% CI	*P* value
Group, the experimental group /control			0.925
T stage, T1 ~ 3/T4	15.071	1.809–125.553	0.012
Histological diagnosis, Well and intermediately differentiated/poorly differentiated			0.360
Change of tumor markers, Decrease and stable/increase			0.167
N stage, N0/N1–3			0.122
P stage, 1–2/3			0.076

## Discussion

Peritoneal recurrence often occurs in gastric cancer patients after radical gastrectomy. Prognosis of the patient with peritoneal recurrence is very poor, median survival time of which is less than 7 months [24]. During the progression of locally advanced gastric cancer, cancer cells are likely to seed on the peritoneum that results in peritoneal dissemination after they invade through serosal layer of gastric wall [25]. Therefore, the deeper the depth of gastric cancer invasion, the more likely to occur peritoneal metastasis or recurrence. HIPEC is an effective method can be used to treat peritoneal metastasis of some cancers. Whether HIPEC can be used to prevent or delay peritoneal recurrence of locally advanced gastric cancer, especially when cancer cells have infiltrated into serous layer of gastric wall, is still obscure. This study was a randomized controlled trial to clarify the safety and tolerability of HIPEC after curative resection for locally advanced gastric cancer. Results showed that administration of HIPEC with cisplatin was safe and well tolerated with low complications and short duration of hospitalization. The thermal effect of HIPEC can enhance the cytotoxicity of chemotherapeutic agents [26–28]. However, combination of thermal effects and drug toxicity can lead to more side effects. The most common complications in the literature are anastomotic leakage, bone marrow suppression and wound infection. In our study, anastomotic or intestinal leakage occurred in 5 patients, 1 in control group and 4 in experimental group. Four patients showed grade II bone marrow suppression, 2 in each group. No would infection occurred in either group. There was no marked difference between the occurrence rates of postoperative complications. Prophylactic HIPEC with radical D2 gastrectomy was relatively safe for locally advanced gastric cancer.

Previous studies that investigated the role of HIPEC for preventing peritoneal recurrence in locally advanced gastric cancer have shown contradictory findings. Kang LY from Taiwan reported that radical gastrectomy with HIPEC had a better prognosis for locally advanced gastric cancer [29]. The 3-year DFS was 28.87% for patients who received surgery, while up to 66.03% for patients underwent surgery followed by HIPEC. The cancer recurrence was delayed in patients treated with HIPEC. In a recent study, Reutovich MY showed HIPEC with cisplatin and doxorubicin reduced metachronous peritoneal metastasis in radically operated serosa-invasive gastric cancer patients [30]. Another randomized study reported that prophylactic HIPEC with radical surgery improved survival and decreased incidence of peritoneal recurrence of advanced gastric cancer [18]. On the other hand, the PHOENIX-GC trial failed to show a survival benefit of intraperitoneal paclitaxel plus systemic chemotherapy in gastric cancer with peritoneal metastasis [31]. In the trial JCOG9206–2, adjuvant chemotherapy with intraperitoneal and intravenous cisplatin after radical gastrectomy did not improve patient overall and relapse free survival for patients with serosa-positive gastric cancer [32]. It should be noted that only 39% of patients who were treated with adjuvant chemotherapy actually completed the regime. Some patients have undergone radical gastrectomy with D2+ lymphadenectomy. For these two reasons, a possible effect of intraperitoneal chemotherapy as prophylaxis against peritoneal recurrence could not be excluded.

In the current trial, HIPEC did not improve the patient survival. In the multivariate survival analysis, only pathological T stage was significantly correlated with prognosis in locally advanced gastric cancer patients who underwent radical resection. HIPEC with cisplatin did not prolong patient survival in locally advanced gastric cancer. Our results are not consistent with Reutovich MY’s results though the research design of these two studies was similar [33]. Patients in HIPEC group of the two studies were both treated with curative resection followed by intraoperative HIPEC with cisplatin. However, there were more stage III patients enrolled in Reutovich MY’s study and the patients were treated with HIPEC for a longer time (60 min) when compared with those in our study. A higher tumor stage indicates a higher probability of peritoneal recurrence and metastasis. The prolonged perfusion time could increase the antitumor efficacy of HIPEC. Taken together, these two studies imply that the perfusion time and selection of patients might affect the efficacy of HIPEC with cisplatin in the prevention of peritoneal recurrence. Interestingly, in our study, the percentage of patients with elevated tumor markers was significantly lower in the HIPEC group than in the control group. Since serum level of tumor markers can be used to monitor recurrence of cancer, these data suggest that HIPEC might inhibit the peritoneal recurrence.

This study has some limitations. In this randomized trial, only 50 patients were enrolled, the sample size may not be big enough to reach a statistically significant difference when comparing the HIPEC group with the control group (type-2 error). Secondly, this was a prospective controlled study. The inclusion criteria of locally advanced gastric cancer patients were based on clinical staging. CT scans were performed for the T staging. Studies have shown that the diagnostic accuracy of CT in determining T stage of gastric cancer was about 73.8–82.7% [34, 35]. Due to the limitation of technology, in this study, 4 patients with T1 disease were over-staged as advanced gastric cancers. These patients were less likely to have peritoneal metastasis or cancer recurrence after radical surgery. This might have influenced the results of the study.

## Conclusion

Prophylactic HIPEC with cisplatin was safe and well tolerated in patients with locally advanced gastric cancer. The survival benefit of HIPEC as a prophylactic therapy for locally advanced gastric cancer patients should be validated by prospective trials in a larger cohort.

## Data Availability

The datasets used and analyzed during the current study available from the corresponding author on reasonable request.

## Abbreviations

HIPEC: Hyperthermic intraperitoneal chemotherapy
RFS: Relapse free survival
BSA: Body surface area
Alb: Albumin
KPS: Karnofsky performance score
BMI: Body mass index
MSS/MSI: Micro-satellite stable/instable
